# A Novel Variant of the FERMT3 Gene Associated With Leukocyte Adhesion Deficiency Type III (LAD-III) in a Saudi Family: A Case Series

**DOI:** 10.7759/cureus.78796

**Published:** 2025-02-09

**Authors:** Badriah G Alasmari, Somayah A Alghubishi, Syed Rayees, Arwa S AlDahmashi, Sami S Alamri, Daifulah AL Zahrani

**Affiliations:** 1 Department of Pediatrics, Armed Forces Hospital Southern Region, Khamis Mushait, SAU; 2 Department of Hematology, Armed Forces Hospital Southern Region, Khamis Mushait, SAU; 3 Department of Pediatrics, King Saud Bin Abdulaziz University for Health Sciences, Jeddah, SAU

**Keywords:** autosomal recessive inheritance, bleeding disorder, consanguinity, fermt3 mutation, immune deficiency, lad-iii, whole-exome sequencing

## Abstract

Leukocyte adhesion deficiency type III (LAD-III) is a rare autosomal recessive disorder characterized by immune dysfunction and bleeding tendencies. The condition arises from mutations in the FERMT3 gene, which disrupts integrin activation on leukocytes and platelets. This case study focuses on a family with consanguineous parents and multiple affected individuals spanning two generations, all diagnosed with LAD-III due to a novel homozygous mutation in the FERMT3 gene (c.1683-22_1683-19del). Clinical manifestations ranged from mild ecchymosis to severe bleeding necessitating transfusions. The proband, a two-year-old male child, presented with recurrent ecchymosis, neonatal sepsis, and thrombocytopenia. His laboratory results included leukocytosis and microcytic hypochromic anemia with normal coagulation profiles. The diagnosis of LAD-III was confirmed through whole exome sequencing that identified the homozygous FERMT3 mutation. Additionally, the proband's 15-year-old sister, who had been earlier diagnosed with Glanzmann thrombasthenia, was found to carry the same mutation, as were the proband’s cousin and the cousin of his father.

## Introduction

Primary immunodeficiency disorders (PIDs) represent a group of rare diseases, with a global prevalence estimated between 0.02% and 0.1%. More than 400 types of PIDs have been documented thus far. The clinical features associated with these disorders are typically non-specific and may overlap with those of malignancies, allergies, inflammatory diseases, autoimmune conditions, and infections [1]. The similarity of these symptoms combined with a widespread lack of knowledge about these rare conditions often leads to diagnostic oversights, which can impede timely and effective patient management [2]. Leucocyte adhesion deficiency (LAD) is a type of PID that occurs when leucocytes have no adhesion molecules. This leads to a failure in their ability to move to areas of infection and inflammation causing repeated serious infections, especially during early childhood, which can result in failure to thrive and even death if not identified and treated promptly [3,4].

LAD is classified according to the mutated gene, with type I linked to ITGB2, type II to SLC35C1, and type III to FERMT3. A new classification, LAD type IV-CFTR, due to pathogenic mutations in the CFTR gene has been identified [5-7]. The FERMT3 gene produces Kindlin-3, a protein essential for integrin activation and the adhesion of various immune cells, including granulocytes, platelets, and lymphocytes. In cases of LAD-III, mutations in the FERMT3 gene result in the inhibition of integrin function [8,9]. This type is marked by atypical integrin activation during the second phase of the adhesion process. Symptoms in LAD-III patients often include recurrent severe infections, a predisposition to leukocytosis, and bleeding, with abnormal platelet function being a key factor in bleeding incidents [10,11]. Some patients may also show radiographic features consistent with osteopetrosis [6]. Diagnosis relies on genetic studies, particularly examining the FERMT3 gene for mutations indicative of LAD-III. Treatment typically involves blood transfusions and antibiotics to manage infections, while the only curative option is bone marrow transplantation [12]. This case study describes a family with four members diagnosed with the LAD-III phenotype due to a newly identified mutation in the FERMT3 gene.

## Case presentation

We observed consanguineous parents and multiple affected individuals across two generations. Four family members presented with clinical features consistent with LAD-III, varying in severity from mild ecchymosis to significant bleeding requiring transfusions. A detailed description of each case is provided below.

Patient 1: Proband

The proband, a two-year-old male child, was referred from Urology for clearance to undergo hypospadias repair. His clinical presentation included recurrent ecchymosis, first noted by his mother at 15 months of age, which resolved spontaneously. He was born at full term to consanguineous parents and required a short neonatal intensive care unit (NICU) stay due to neonatal sepsis and thrombocytopenia. At six months, he experienced recurrent bronchiolitis but had no significant bleeding issues such as epistaxis, bloody diarrhea, or joint swelling. The examination was unremarkable apart from ecchymosis and hypospadias. Laboratory findings, including coagulation profile and von Willebrand factors, were normal. Whole exome sequencing (WES) recognized a homozygous FERMT3 mutation (c.1683-22_1683-19del), leading to a diagnosis of LAD-III. Consequently, he was referred for an immunological evaluation and preparation for a potential bone marrow transplant. Laboratory details are provided in Table 1.

**Table 1:** Complete blood count values

Parameter	Normal range	Patient 1	Patient 2	Patient 3	Patient 4
White blood cells (WBCs)	4-13 x 10^9^/L	34.33	15	26.5	16
Red blood cells (RBCs)	4.1-5.3 x 10^6^/µL	4.75	3.7	4	3.4
Hemoglobin	10.9-15 g/dL	10.8	7	9	7
Hematocrit (HCT)	31%-41%	36.2	21	30.6	22
Mean corpuscular volume (MCV)	73-89 fL	76.1	59	76.6	57
Mean corpuscular hemoglobin (MCH)	23-30 pg	22.7	15	22.6	14.3
Mean corpuscular hemoglobin concentration (MCHC)	32-36 g/dL	29.8	25	29.5	24.7
Red cell distribution width (RDW)	11%-14%	21.7	22	19.5	21.3
Platelet count (PLT)	150-450 x 10^9^/L	497	150	336	167
Mean platelet volume (MPV)	9.4-12.3 fL	9.0	8	10.2	9.8
Absolute lymphocyte count (LYMPH ABS)	4-10.5 x 10^9^/L	23.44	6.2	17.59	6

Peripheral blood analysis displayed hypochromic microcytic red cells with leukocytosis, mature-looking lymphocytes with occasional large mononuclear cells, mostly activated lymphocytes, and adequate platelets. Other laboratory tests including coagulation profile, Protein C and S, and von Willebrand factor (vWF) levels were normal.

**Figure 1:**
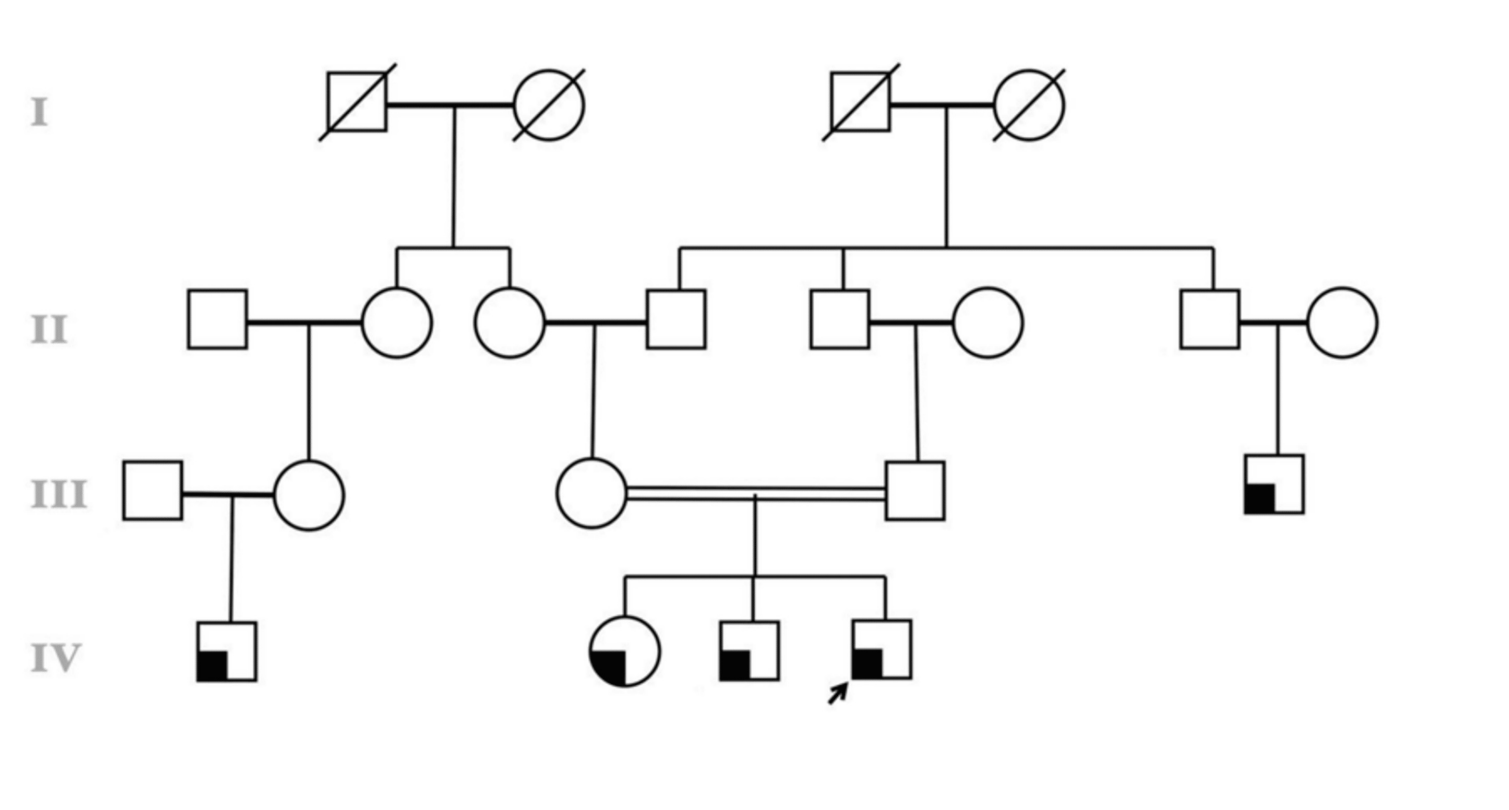
Family pedigree Shaded symbols represent the affected family members. The proband (generation IV affected individual) is indicated by an arrow.

The pedigree chart presented in Figure 1 shows an autosomal recessive mode of inheritance in a consanguineous family. Shaded symbols represent the affected individuals, seen mostly in the fourth generation, suggesting that the recessive condition is more likely to manifest in offspring of consanguineous unions, as suggested by the double horizontal lines connecting related individuals in generation III. The proband (generation IV affected individual) case served as the rationale for the genetic investigation. The elder brother of the proband had very mild symptoms of occasional epistaxis.

Patient 2: Sibling

Patient 2, a 15-year-old female (older sister to patient 1) was diagnosed with Glanzmann thrombasthenia at the age of 2 after recurrent ecchymosis. Her medical history included delayed cord separation at 14 days, BCGitis at two months, and recurrent chest infections. At the age of 11, she started to bleed heavily. No significant findings were noted on examination. The coagulation profile and vWF levels were normal in laboratory studies. A complete blood count for the patient is shown in Table 1. The peripheral smear revealed hypochromic microcytic severe anemia with polychromasia and normal platelets. The homozygous FERMT3 mutation was confirmed in her by WES.

Patient 3: Cousin

The patient was a three-year-old male child, a cousin of patient 1, with a history of recurrent ecchymosis, and recurrent episodes of bronchiolitis. Epistaxis and separation of the cord stump occurred at 12 days of age. The patient's mother also noticed that the recurrent episodes of ecchymosis in the lower legs of the patient went away without treatment. Café-au-lait spots, ecchymosis in the lower limbs, and hypospadias were the only physical findings. Complete blood count (Table 1) testing showed leukocytosis with predominant lymphocytes. Peripheral blood analysis showed hypochromic microcytic and occasional polychromasia with Rouleaux formation, consistent with iron deficiency anemia. WBC analysis revealed lymphocytosis (75%). WES identified a homozygous variant (c.1683-22_1683-19del) of uncertain significance in the FERMT3 gene. This case was published as a case report in 2023 [6]. Following the diagnosis of the proband through WES, genetic analyses were conducted on additional members of the extended family, revealing the presence of the same novel mutation in all individuals tested.

Patient 4: Father's cousin

Patient 4 was an 18-year-old male, and a cousin of the father of patient 1 (proband); he had a history of recurrent gum bleeding that required blood and platelet transfusions. At the age of 7, he was initially diagnosed with von Willebrand disease type 1, but a recent laboratory evaluation showed normal vWF levels, ruling out the diagnosis. His complete blood count values are mentioned in Table 1. The same homozygous FERMT3 mutation was found by WES in this case. Currently, he is stable without active bleeding episodes.

Genetic testing of all patients

Whole exome sequencing revealed a novel homozygous variant (c.1683-22_1683-19del) in the FERMT3 gene in all four family members. This mutation, which has not been reported in ClinVar or other genetic databases, is predicted to cause exon skipping, resulting in an abnormal protein product. The mutation is associated with LAD-III phenotypes, characterized by bleeding tendencies and recurrent infections. Both parents were confirmed as carriers of the mutation, consistent with the autosomal recessive inheritance pattern. The family’s consanguinity likely contributed to the expression of the mutation in multiple members. A brief clinical presentation of all cases is provided in Table 2.

**Table 2:** Brief clinical presentation of the studied cases vWF, von Willebrand factor

Parameter	Patient 1 (proband)	Patient 2, sister	Patient 3, cousin	Patient 4, father’s cousin
Age at diagnosis	2 years	15 years	3 years	18 years
Presenting symptoms	Recurrent ecchymosis, recurrent bronchiolitis, hypospadias	Recurrent ecchymosis, BCGitis, recurrent chest infections, menorrhagia	Recurrent ecchymosis and bronchiolitis, epistaxis, hypospadias, café-au-lait spots	Recurrent gum bleeding requiring transfusion
Cord separation	Cord separation at 15 days of age	Cord separation at 14 days of age	Cord separation at 12 days of age	Delayed separation around second week of life
Diagnosed before with	Nil	Glanzmann thrombasthenia	Nil	von Willebrand disease type 1
Blood parameters	Leukocytosis	Thrombocytopenia	Lymphocytosis	Leukocytosis
Coagulation parameters	Normal coagulation profile and vWF levels	Normal coagulation profile and vWF levels	Normal	Normal coagulation profile and vWF levels
Genetic diagnosis	FERMT3 mutation	FERMT3 mutation	FERMT3 mutation	FERMT3 mutation

## Discussion

LAD-III patients often experience recurrent non-purulent infections and a bleeding condition similar to Glanzmann thrombasthenia, attributed to a malfunction in platelets. LAD-III is a condition characterized by joint defects, similar to LAD-I (MIM#116920) [13]. The defect affects an intracellular protein that interacts with β-integrins in hematopoietic cells, including platelets, macrophages, lymphocytes, and granulocytes [14]. The inability to activate β1 and β2 integrins on granulocytes and lymphocytes, and β3 integrin on platelets results in its main clinical features [13].

Integrins αIIbβ3 and α2β1 are responsible for irreversible platelet adhesion to collagen and vWF and promote platelet spreading and recruitment [15]. Integrin β1 (CD29) forms heterodimers with various α subunits such as α5β1 (a fibronectin receptor), α6β1 (a laminin receptor), and α4β1 (a receptor for vascular cell adhesion protein 1), and these heterodimers are critical for cell interactions [16]. In contrast, β2 integrin (CD18) is expressed uniquely in leukocytes. It also facilitates cell interactions (e.g., binding to intercellular adhesion molecule 1, or ICAM1, on activated endothelial cells, thereby mediating neutrophil spreading and adhesion) when paired with specific α subunits (such as αM or CD11b) [15].

Integrin β3 (CD61) also pairs with the αv subunit to form the αvβ3 receptor that binds vitronectin and is involved in cell adhesion, spreading, and signaling. Key features of LAD-III, including granulocytosis, lymphocytosis, and life-threatening mucocutaneous bleeding are accounted for by the defective "inside-out" signaling [17]. LAD-III clinical manifestations are underscored by these immune and hemostatic failures. In our case, the sibling of the first patient was diagnosed with Glanzmann thrombasthenia, and patient 3 had lymphocytosis.

Regarding clinical features of LAD-III, in a study, umbilical cord separation was noted at 12 days, which exceeded the standard separation time (mean ± SD: 6.61 ± 2.33 days). Clinically, this patient presented with a history of recurrent ecchymosis, bleeding episodes, and repeated episodes of bronchiolitis [18]. These findings are consistent with the typical manifestations observed in our LAD-III patients, such as ecchymosis, bleeding episodes, umbilical cord separation at 12 days, and bronchiolitis.

The differential diagnosis for leukocytosis in LAD-III includes infection, and leukemoid reaction (leukocytosis with circulating immature white and red blood cells). This can sometimes resemble chronic myelogenous leukemia, chronic myelomonocytic leukemia, and lymphoproliferation, which includes leukemia. Also, eosinophilia might be mimicking an atopic condition. A T-cell lymphoma can look similar to a large thymus. However, defined increases in granulocytes and lymphocytes remain, suggesting a leukocyte adhesion defect [19,20]. LAD-III should typically be distinguished from LAD-I, leukemoid reactions, and Glanzmann thrombasthenia.

The treatment for LAD-III focuses on supportive care, which involves topical thrombin and an anti-fibrinolytic agent like aminocaproic acid or tranexamic acid. It is important to monitor how the body responds to anti-fibrinolytic agents and to adjust the dosage if needed. Platelet transfusion is used in cases of severe bleeding, or can be given weekly as a preventive measure when needed. It is important to take precautions when it comes to circumcision and maintain skin and mucosal hygiene, including dental care. It is also crucial to start antimicrobial therapy quickly if there is an infection since fever and inflammatory biomarkers might not always be obvious or noticeable. Prophylactic treatment for pneumocystis pneumonia, usually with trimethoprim/sulfamethoxazole, is recommended [19].

Therapeutically, patients face challenges requiring prophylactic antibiotics and frequent blood transfusions, with hematopoietic stem cell transplantation (HSCT) as the curative option. The requirement for transfusions might rise dramatically, topping 20 or 50 transfusions annually for platelets and erythrocytes, respectively [16]. Some cases have even granulocyte transfusions to improve pathogen clearance. Individuals with LAD-III who have not had transplantation have a low survival rate, which is exacerbated by high mortality, as indicated by deceased siblings who had similar symptoms but were not diagnosed. According to research, less than four people have survived childhood without HSCT, and the recorded maximum age is 20 years, requiring periodic platelet transfusions. Successful HSCT can provide a life free of symptoms; however, the technique is hampered by pretransplant infections, bleeding issues, and potential complications caused by osteopetrosis during the conditioning regimen. Despite an increase in HSCT success rates, addressing these problems remains an important element of treatment plans for patients with LAD-III [18-19].

The mutation identified in this study, c.1683-22_1683-19del, is novel and, to our knowledge, has not been previously reported in genetic databases like ClinVar, making this a unique case. Similar FERMT3 mutations have been identified in other cases of LAD-III, but most are located in different regions of the gene. The exon skipping is thought to impair the FERMT3 protein's function in leukocyte adhesion, which is essential for immune cell migration and response to infections.

## Conclusions

In this case study, we described a novel homozygous mutation in the FERMT3 gene (c.1683-22_1683-19del) in a family with LAD-III, an autosomal recessive disorder with immune dysfunction and bleeding tendencies. Affected individuals included those with mild ecchymosis and those with severe bleeding episodes requiring transfusion, as well as recurrent infections and thrombocytopenia. The mutation was confirmed by whole exome sequencing in multiple family members, consistent with an autosomal recessive inheritance pattern, and both parents were identified as carriers. This case reinforces the need for genetic testing, including WES, in making the diagnosis of rare and complex genetic disorders, such as LAD-III, in consanguineous families where autosomal recessive conditions are more likely to be expressed and in pediatric patients presenting with unexplained bleeding disorders. The recognition of novel genetic variants and the identification of diverse clinical phenotypes add to the collective understanding that can shape future research and clinical methodologies.
